# McCune-Albright syndrome – A case report with transmission electron microscopy^☆^

**DOI:** 10.1016/j.abd.2021.09.002

**Published:** 2021-11-25

**Authors:** Victor Garcia Neto, Hiram Larangeira de Almeida Jr, Claúdia Fernandes Lorea, Valéria Magalhães Jorge, Antônia Larangeira de Almeida

**Affiliations:** a Post-Graduation Program in Health Sciences, Universidade Católica de Pelotas, Pelotas, RS, Brazil; b Department of Dermatology, Universidade Federal de Pelotas and Universidade Católica de Pelotas, Pelotas, RS, Brazil; c Genetics, Universidade Federal de Pelotas, Pelotas, RS, Brazil; d Adjunct Professor of Pathology, Universidade Federal de Pelotas, Pelotas, RS, Brazil; e Dermatology League, Universidade Federal de Pelotas, Pelotas, RS, Brazil

**Keywords:** Cafe-au-lait spots, Fibrous dysplasia, polyostotic, Microscopy, electron, transmission

## Abstract

McCune - Albright syndrome is a genetic disease with cutaneous mosaicism caused by post-zygotic activating mutations in GNAS locus, it has a triad of fibrous bone dysplasia, café-au-lait macules and precocious puberty. We examined a 22-year-old female patient with café au lait spot in right side of the abdomen, with a chessboard - like distribution, extending to right thigh with geographical contours, she has also an ovarian cyst, scoliosis and truncal obesity. Biopsies were taken from the hyperpigmented area and processed for light microscopy and for transmission electron microscopy. Light microscopy showed increased melanin pigment with HE staining. Immunohistochemistry with melanocytic markers (HMB-45 and Melan-A) revealed a normal number of melanocytes. Transmission electron microscopy demonstrated normal epidermal structures, such as desmosomes, cytokeratin filaments and hemidesmosomes. With high magnifications an irregular melanossomal contour was seen, with some indentations in their outline.

## Introduction

McCune*-Albright Syndrome (MAS) – OMIM* #174800 – is a genetic disease with cutaneous mosaicism caused by post-zygotic activating mutations in GNAS locus, encoding G_s_α protein.^1^, ^2^ MAS was originally described in 1936 as a triad of fibrous dysplasia of bone, *café-au-lait* skin macules, and precocious puberty. However, it is now recognized that its phenotype is far more complex.^3^ Other endocrinopathies, including hyperthyroidism, estrogen-producing ovarian cysts, growth hormone excess, renal phosphate wasting with or without rickets/osteomalacia, and Cushing syndrome could be seen in association with the original triad.^2^, ^4^

MAS is most often identified by dermatologic findings. *Café-au-lait* macules appear in the neonatal period or in the first months of life. These macules have irregular borders (described as “coast of Maine”) and are unilateral.^5^

There are yet substantial knowledge gaps about MAS pathophysiology and natural history^6^ and very little information about its *café-au-lait* macules, which were examined by light microscopy, immunohistochemistry, and Transmission Electron Microscopy (TEM) in this report.

## Materials and methods

**Figure 1:**
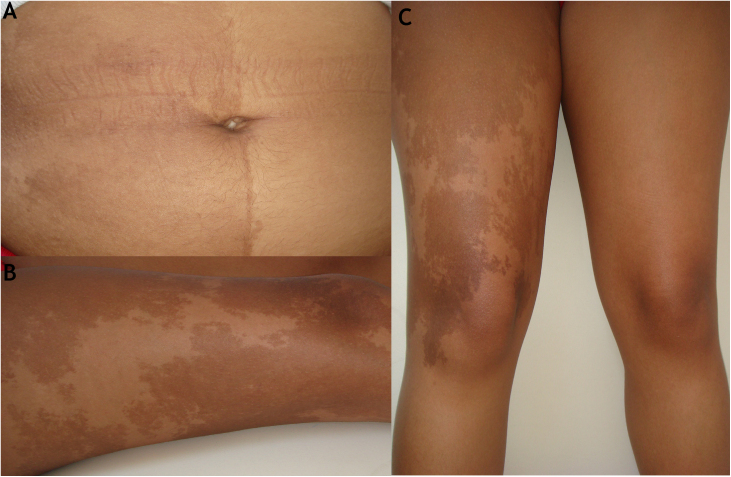
(A), Segmental hyperchromic *café-au-lait* spot in the abdomen, with a chessboard-like distribution, respecting the midline of the body; (B and C), Segmental hyperchromic *café-au-lait* in the right thigh with geographical contour.

The authors examined a 22-year-old female patient who presented since birth with segmental hyperchromic *café-au-lait* spots in the right side of the abdomen, with a chessboard-like distribution, respecting the midline (Fig. 1a), extending to the right thigh with geographical contours (Figs. 1b and c). The patient was submitted to surgical correction of scoliosis in childhood and presented also an ovarian cyst and truncal obesity. She had been diagnosed with neurofibromatosis type 1 in her childhood, however, without the presence of neurofibromas, Lish nodules, or axillary ephelides.

Biopsies were taken from the hyperpigmented area and processed for light microscopy (hematoxylin-eosin and immunohistochemistry with HMB-45 and Melan-A) and for TEM, in the latter, ultrathin sections targeted the epidermis.

## Results

**Figure 2:**
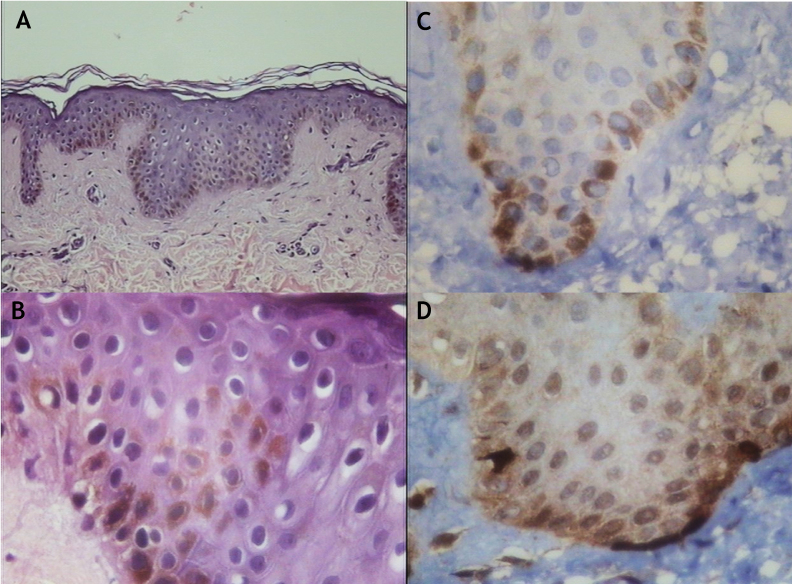
Light microscopy. (A), Increased melanin pigment with hematoxylin and eosin staining (×150). (B), Increased melanin pigment with hematoxylin and eosin staining which was also seen in suprabasal layers (×400). (C), Immunohistochemistry with melanocytic marker (Melan A) confirmed an increase of melanin and revealed a normal number of melanocytes (×400). (D), Immunohistochemistry with melanocytic marker (HMB-45) confirmed an increase of melanin and revealed a normal number of melanocytes (×400).

Light microscopy showed increased melanin pigment with HE is staining (Fig. 2a), in some areas the pigment was seen also in suprabasal layers (Fig. 2b). Immunohistochemistry with melanocytic markers (HMB-45 and Melan-A) revealed a normal number of melanocytes (Figs. 2c and d).

**Figure 3:**
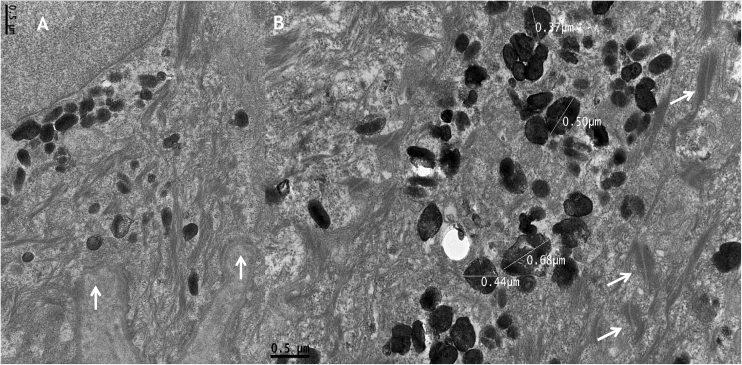
Transmission electron microscopy (A), basal keratinocyte with hemidesmosomes and normal basement membrane (arrows) (×30,000). (B), Large amount of heterogen melanosomes with normal size and normal desmossomes (arrows) (×30,000).

**Figure 4:**
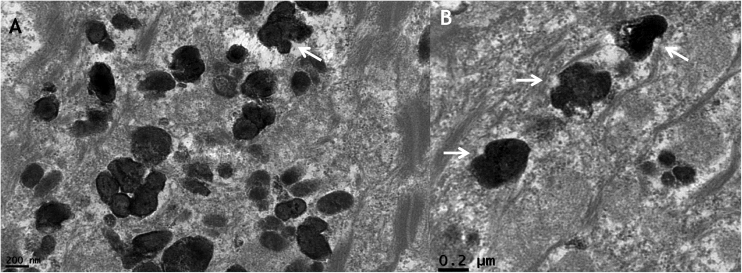
Transmission electron microscopy (A and B). Detail of melanosomes with indentations in their outline (×50,000).

**Figure 5:**
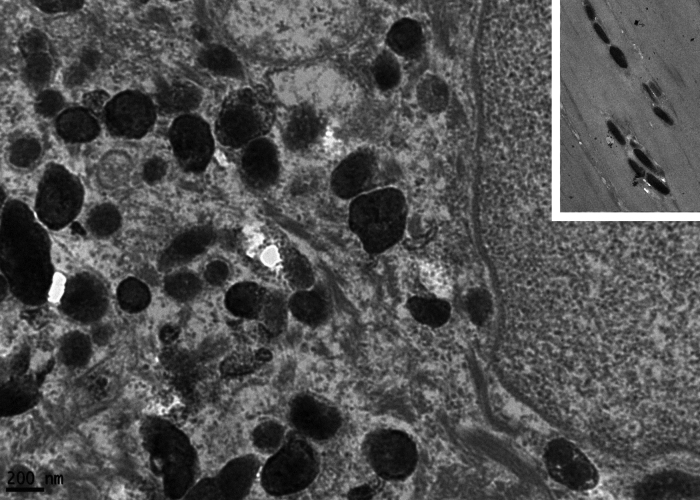
Transmission electron microscopy – detail of irregular melanosomes, inset with normal regular oval melanossomes (×50,000).

TEM demonstrated normal epidermal structures, such as desmosomes, cytokeratin filaments, basement membrane and hemidesmosomes (Figs. 3a and b). A high amount of melanosomes in keratinocytes was observed (Fig. 3b), which measured 0.38 to 0.67 nm, a variation in the form and size could also be observed (Figs. 3b, 4a and 5).

With high magnifications indentations in melanosomes’ outline (Fig. 4a and b) could be seen, as well as an irregular melanosomal contour was observed (Fig. 5), in contrast to normal melanosomes, which show a regular contour and an oval shape (Fig. 5 inset).

## Discussion

The clinical signs of the examined patient are in accordance with the literature, with the segmental *café-au-lait* macule, bone, and ovarian involvement.

Light microscopy observed an increase in the melanin pigment with a normal number of melanocytes in hyperchromic MAS lesions, showed by melanocyte markers.

There are no reports of ultrastructural studies in *café-au-lait* macules of MAS, the authors found only one study that evaluated *café-au-lait* spots using electron microscopy in neurofibromatosis in 1992. No abnormal ultrastructural findings were observed in the melanocytes or epidermal keratinocytes, melanosomes (either in melanocytes or in epidermal keratinocytes) had a normal aspect.^7^

The authors’ findings have also demonstrated a similar pigment increase. With a high-power view, however, the melanosomes present an abnormal aspect, suggesting a defect in their synthesis in MAS.

This study presents the first description of the electron microscopic features of *café-au-lait* spots in a patient with MAS, describing ultrastructural changes in melanosomes, which appeared with an irregular outline in high magnifications. Dermatologists should recognize this disorder, given the possibility that these macules seen in MAS can be confused with neurofibromatosis, as in the case described here.

## Financial support

None declared.

## Authors’ contributions

Victor Garcia Neto: Approval of the final version of the manuscript; design and planning of the study; drafting and editing of the manuscript; collection, analysis, and interpretation of data; critical review of the manuscript.

Hiram Larangeira de Almeida Jr.: Approval of the final version of the manuscript; design and planning of the study; drafting and editing of the manuscript; collection, analysis, and interpretation of data; effective participation in research orientation; critical review of the manuscript.

Claúdia Fernandes Lorea: Approval of the final version of the manuscript; design and planning of the study; drafting and editing of the manuscript; collection, analysis, and interpretation of data; critical review of the manuscript.

Valéria Magalhães Jorge: Approval of the final version of the manuscript; design and planning of the study; drafting and editing of the manuscript; collection, analysis, and interpretation of data; critical review of the manuscript.

Antônia Larangeira de Almeida: Approval of the final version of the manuscript; design and planning of the study; drafting and editing of the manuscript; collection, analysis, and interpretation of data; critical review of the manuscript.

## Conflicts of interest

None declared.
